# Multi-Response Optimization of Tensile Creep Behavior of PLA 3D Printed Parts Using Categorical Response Surface Methodology

**DOI:** 10.3390/polym12122962

**Published:** 2020-12-11

**Authors:** Muhammad Waseem, Bashir Salah, Tufail Habib, Waqas Saleem, Muhammad Abas, Razaullah Khan, Usman Ghani, Muftooh Ur Rehman Siddiqi

**Affiliations:** 1 Department of Industrial Engineering, University of Engineering & Technology, Peshawar 25100, Pakistan; mw9722299@gmail.com (M.W.); tufailh@uetpeshawar.edu.pk (T.H.); 2 Industrial Engineering Department, College of Engineering, King Saud University, P.O. Box 800, Riyadh 11421, Saudi Arabia; bsalah@ksu.edu.sa; 3 Department of Mechanical and Manufacturing Engineering, Institute of Technology, F91 YW50 Sligo, Ireland; waqas95@yahoo.com; 4 Department of Mechanical Engineering Technology, University of Technology, Nowshera 24100, Pakistan; 5 Department of Mechanical Engineering, University of Engineering and Technology, Peshawar, Jalozai Campus 24240, Pakistan; usmanghani@uetpeshawar.edu.pk; 6 Department of Mechanical Engineering, CECOS University of IT and Emerging Sciences, Peshawar 25100, Pakistan; muftooh@cecos.edu.pk

**Keywords:** fused deposition modeling, polylactic acid, tensile creep behavior, modeling polymer manufacturing, design for additive manufacturing

## Abstract

Three-dimensional printed plastic products developed through fused deposition modeling (FDM) endure long-term loading in most of the applications. The tensile creep behavior of such products is one of the imperative benchmarks to ensure dimensional stability under cyclic and dynamic loads. This research dealt with the optimization of the tensile creep behavior of 3D printed parts produced through fused deposition modeling (FDM) using polylactic acid (PLA) material. The geometry of creep test specimens follows the American Society for Testing and Materials (ASTM D2990) standards. Three-dimensional printing is performed on an open-source MakerBot desktop 3D printer. The Response Surface Methodology (RSM) is employed to predict the creep rate and rupture time by undertaking the layer height, infill percentage, and infill pattern type (linear, hexagonal, and diamond) as input process parameters. A total of 39 experimental runs were planned by means of a categorical central composite design. The analysis of variance (ANOVA) results revealed that the most influencing factors for creep rate were layer height, infill percentage, and infill patterns, whereas, for rupture time, infill pattern was found significant. The optimized levels obtained for both responses for hexagonal pattern were 0.1 mm layer height and 100% infill percentage. Some verification tests were performed to evaluate the effectiveness of the adopted RSM technique. The implemented research is believed to be a comprehensive guide for the additive manufacturing users to determine the optimum process parameters of FDM which influence the product creep rate and rupture time.

## 1. Introduction

With the advent of reliable polymeric materials and advanced printing techniques, additive manufacturing (AM) has emerged an indispensable pillar in the fabrication of simple to complex products [1,2,3]. Among various additive manufacturing technologies, fused deposition modeling (FDM) outweighs the other techniques due to its simple processing and economical applications [1,2,3,4]. FDM printer uses thermoplastics filaments which are fed into an extruder and subsequently melted by the heater. The molten material is then extruded through a nozzle to develop a multi-layer platform in the form of the desired shape. The commonly used thermoplastics are acrylonitrile butadiene styrene (ABS), polylactic acid (PLA), nylon, and fiber-reinforced composites [1,5]. These materials are economical, lightweight, and exhibit excellent properties.

There are four major crystalline forms (α, β, γ, and δ) which are mostly exhibited by PLA [6,7,8,9,10]. The common α crystallite form is obtained from the melt in a slow cooling procedure which allows the PLA chain to rotate into the confirmation with lower potential energy. The β crystalline phase arises from a deformation of the crystals and is usually obtained by drawing the PLA at elevated temperatures; the γ form is obtained by epitaxial crystallization on a single substrate, such as hexamethylbenzene [10]. Due to the similarity in the crystalline structure, δ form is also called α’, or imperfect α form [11]. The δ form (or α’ form) is observed in samples processed from the melt with a fast-cooling procedure [12]. The crystallization of PLA at high super cooling of the melt leads to the formation of the disordered conformation, i.e., δ crystals. These crystals are metastable both at the temperature of their formation and below. From the melt, the polymer tends to crystallize in α form but actually also partially crystallizes in α’ form at lower temperatures. In this case, the PLA has higher internal stresses and presents a lower thermal and mechanical stability. Considering the effect of the printing pathway, as well, the printed part is always anisotropic.

Cicala et al. [13] tested three commercial PLA filaments and compared their properties using thermos gravimetric analysis (TGA) and scanning electron microscopy (SEM) analysis. Two filaments (White and Black) showed better results in terms of rheology, while the other one (Green) resulted in poor rheological properties. Overall, the filament filled with mineral fillers showed the best thermomechanical performance and printing quality. Cicala et al. [13] prepared two blends of Polyetherimide (PEI) by modification with Polycarbonate (PC) and polyethylene terephthalate glycol-modified (PETG). They compared the blends with Ultem 9085, a standard grade for FDM printing, and analyzed different properties like tensile and thermomechanical properties. The PC blend showed good results with low PC content, but the properties were not satisfactory with higher PC content. On the other hand, the PETG blend showed significant results with almost similar properties to the Ultem 9085.

Olaiya et al. [14] analyzed the mechanical, thermal, and microstructural properties of PLA by blending it with chitin. The percentage weight of the chitin was kept from 2 to 8 percent. From the tensile strength, yield strength, Young’s modulus, and impact tests, it was analyzed that the PLA–chitin blend shows excellent mechanical properties. Subramaniam et al. [15] investigated the tensile property of PLA to determine the optimum printing parameter. Tensile specimens were printed according to ASTM D638 type 1 standard and varying the infill density. Three important mechanical properties were investigated, namely ultimate tensile strength, elastic modulus, and yield strength, and it was inferred that the tensile property increases with the infill density.

Getme et al. [16] evaluated that the fragility of PLA can be reduced by reinforcing with other biodegradable products, and it is an effective way to produce completely biodegradable composites. By reducing the brittleness of PLA, it can be used in a broad variety of applications. Cobos et al. [17] proposed PLA as an alternative to nanocomposites in fusion deposition modeling. The work involved describing thermal and rheological conditions of the PLA with multi-wall carbon nanotube (PLA/MWCNT) and halloysite nanotube (PLA/HNT) composites. The research was accomplished by employing differential scanning calorimetry (DSC) and capillary rheometry to characterize these products and analyze the melt flow index (MFI).

PLA applications are also common in pharmaceuticals and healthcare. Juan et al. [18] worked on coating PLA pellets with lignin (LIG) powder and biocompatible oil. The PLA filaments were produced with an extruder at 200 °C. It was found that LIG content affected the mechanical and surface properties of the material and lowered the resistance to fracture. In addition, the resulting 3D printed materials demonstrated antioxidant capabilities. Likewise, DeStefano et al. [19] carried out a detailed study to explain the important applications of PLA in the medical sector, such as tissue engineering, cardiovascular implants, dental implants, orthopedic intercession, tendon healing, and medical equipment. The authors descried that it is a versatile biopolymer and synthesized with ease from abundant renewable resources. Moreover, PLA has successfully used in personal protective equipment (PPE) and ventilator modifications.

Luchian-Lupu et al. [20] evaluated the stability qualification of PLA and styrene-isoprene-styrene triblock polymers (SIS) blend. Samples were prepared with three SIS loadings (10, 20, and 30 percent weight) in the PLA matrix. It was revealed that the concentration effect of minor component reveals the higher stability for the lower amounts of SIS. It was reported that SIS promote material aging. Camargo et al. [21] studied the mechanical properties of 3D-printed parts produced by PLA-graphene, such as tensile strength, flexural strength, and impact energy. The parts were manufactured with FDM technology with varying the infill and layer thickness parameters. It was investigated that the mechanical properties improve as the linear layer thickness parameter increases, whereas the tensile strength and flexural strength increased with an increase in the infill increased.

Parameters setting in FDM play a significant role in the development of products with desired properties. According to Casavola et al. [22], the optimal set of parameters is a serious challenge in FDM, as several significant factors contribute to the mechanical properties. Some of the important parameters are infill percentage, raster angle, layer thickness, infill pattern, raster width, and air gap. Since poor material properties in 3D printing pose major problems, therefore, the effect of mechanical properties should be investigated. Baich et al. [23] studied the effect of infill design on production cost and mechanical strength of ABS 3D-printed parts. They concluded that a solid fill design provides a higher strength (tensile, compression, and bending) compared to a high-density infill design with the same cost of production. Farbman and McCoy [24] studied the effect of infill percentage, infill geometries, and load orientation on the ultimate tensile strength of PLA and ABS 3D-printed parts. They compared the strength to weight ratios of different infill geometries and concluded that the ultimate strength decreases with a decrease in infill percentage, while hexagonal infill patterns are stiffer and stronger than rectilinear infill patterns. According to Caminero et al. [25], the structural properties of FDM are significantly affected by build orientation. Build orientation represents the directions in which the sample is placed on the 3D printer bed surface. They identified that the strongest printing orientation is obtained when the fused fifilament deposition coincided with the pull direction.

Kamoonaet al. [26] studied the effect of build orientation, layer thickness, raster angle, air gap, and raster width on tensile, impact, and flexural strength of FDM-printed parts. They concluded that smaller layer thickness increases the number of layers to fabricate the part; hence it improves the strength of the parts. However, smaller raster angle results in weak bonding layers; therefore, it reduced the strength of the part, while thick raster and zero air gaps result in strong bond formation. Tymrak et al. [27] observed the highest tensile strength for the lowest thickness of L = [0.2, 0.4] mm. However, the authors observed greater variability among parameters for PLA specimens. Pei et al. [28] inferred that increasing the number of shell perimeters improves the relation of tensile strength and layer thickness for PLA specimens. Vaezi et al. [29] reported that, for flflat oriented samples, a decrease in layer height from 0.1 mm to 0.087 mm increased the tensile strength and decreased flflexural strength. According to Lovo et al. [30], optimal build surface orientation is also important to maximize the tensile strength of FDM printed parts.

There are a very limited numbers of publications on creep behavior of PLA parts produced through FDM. This area needs meticulous research efforts in the form of numerical modeling, simulation studies, and experimental validation. Mohamed et al. [31] investigated the flexural creep stiffness behavior of Polycarbonate-ABS (PC-ABS) through definitive screening design. They developed a model with a high coefficient of determination, but this model does not apply to PLA parts; however, it works well with ABS and PC-ABS parts. An et al. [32] predicted the creep behavior of ABS polymeric material using finite element analysis. The numerical outcomes were compared with the experimental and simulation results and found in close approximation with the real-time environment. Lim et al. [33] presented a method for prediction of long term creep behavior using a short term creep experimental data. The experiments were performed on ABS, PC-ABS, and long fiber reinforced thermoplastics (LFT). It was suggested that the method developed for these materials is also applicable to other plastics, like PLA, but the scope of work was very limited. Tezel et al. [34] studied the creep behavior of PLA and PLA composites 3D-printed parts. They concluded that samples built at 90° orientation with a layer thickness of 0.1 mm provide good creep strength. However, it was inferred that the creep behavior can further be explored for possible combinations of printing parameters.

From the presented literature review, it is clear that no comprehensive study is available which shows the creep behavior of PLA 3D-printed parts through FDM. The authors could find only one published research by Tezel et al. [34]. The research was focused on the creep behavior of PLA by undertaking the effect of printing orientation and layer thickness. However, the parameters, such as infill percentage and infill pattern type, were not considered. Moreover, no specific optimization technique was applied to identify the best combination of printing parameters. To fill this gap, the presented research characterizes and optimizes the creep behavior (creep rate and rupture time) of FDM-printed parts under different printing parameters, such as the infill percentage, infill pattern type, and layer thickness. For this aim, response surface methodology is applied to the optimization of printing parameters of creep behavior to predict creep rate and rupture time. Subsequently, ANOVA is applied to study the effect of printing parameters on creep rate and rupture time. The regression model successfully established the relationship between printing parameters and responses. Finally, important confirmation tests are performed to evaluate the adequacy and effectiveness of optimized parameters.

The presented study also provides a comprehensive insight into the influence of FDM process parameters on creep rate and rupture time for the PLA 3D-printed products. This work is particularly interesting for small and medium enterprises in developing countries that are underpinned to adopt additive manufacturing by means of small-scale desktop 3D printers.

## 2. Materials and Methods

### 2.1. Experimental Setup

**Figure 1:**
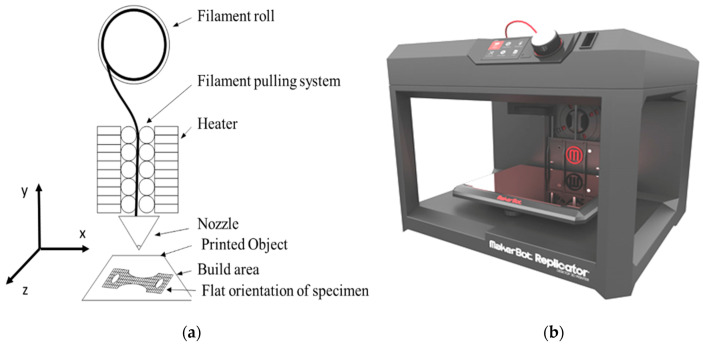
(**a**) Schematic of fused deposition modeling. (**b**) Actual 3D printing setup of fused deposition modeling (FDM).

In the present study, polylactic acid (PLA) specimens are fabricated using a MakerBot desktop 3D printer (MakerBot Industries, New York, NY, USA). Figure 1a shows a schematic of the printer which works on fused deposition modeling (FDM). The object is printed by a continuous supply of melted material through the nozzle. Figure 1b shows the actual printing setup. For creep behavior analysis, specimens were manufactured according to the ASTM D2990 standards. In order to ensure the uniformity of test specimens, each specimen is printed separately by locating it in the middle of the printing bed in a flat orientation. Dave et al. [35] concluded that flat orientation provides good mechanical strength to 3D printed parts through the FDM technique. PLA material is extruded at 240 °C at a speed of 130 mm/s by maintaining the heated bed surface at 70 °C. Commercial-grade PLA (MakerBot Industries, New York, NY, USA), manufactured specifically for the Makerbot printers, was used for this study, which is similar to the material used in biodegradable plastic packaging. It melts between 180 to 200 °C, depending on other material added to it for color and texture. The important material properties of PLA are given in Table 1 [36,37,38].

### 2.2. Experimental Design

Experiments were designed using a categorical central composite design (CCCD) based on Response surface methodology (RSM). The purpose of applying this design is to determine the interaction between the independent variables, model the system mathematically, and to reduce experimental runs [39,40]. It is a second-order fractional factorial design having center points and axial points added to the full factorial design (2^k^), where k is a number of factor, and 2 is the number of levels. Center points indicate the middle level of all factors to be studied and provide an error estimation of experiments, model adequacy checking, and curvature detection in the fitted data [41]. Axial points construct new extreme levels, i.e., low and high for each factor, and provide experimental error assessment [42,43]. In the present study, the CCD comprises of N experimental runs, i.e., N = 2^k^ + 2k + n, where k is the number of continuous numerical factors, 2^k^ is the number of the factorial points at the corners of the cube (2^2^), 2k is the number of the axial points of each numerical factor on the axis at a distance of ±α from the center of the cube (2 × 2), and n is the number of center points (n = 5). As the α value is assumed to be equal to ±1, so the points are at the center of each face of the factorial space, and this type of central composite design is called face-centered central composite design. For one level of a categorical factor, there are 13 experimental runs (N = 2^2^ + (2 × 2) + 5); hence, for three categorical factors, the total experimental runs are 3N (3 × 13 = 39). The continuous factors are layer height (L) and infill percentage (I). The categorical factor is the infill pattern type, i.e., linear, hexagonal, and diamond. Their levels used in the design of experiments are summarized in Table 2. The layer height and the infill percentage varied from 0.1 to 0.3 mm and 10 to 100%, respectively.

**Figure 2:**
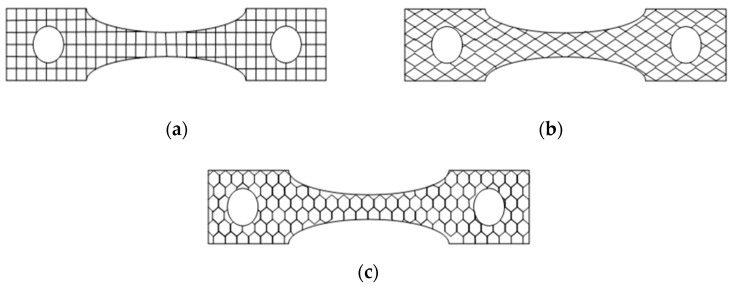
The adopted infill patterns: (**a**) linear, (**b**) diamond, (**c**) hexagonal.

**Figure 3:**
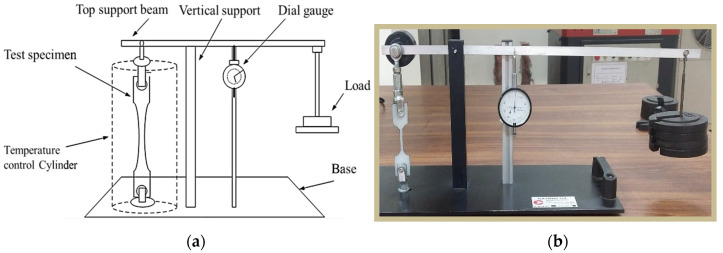
Creep testing machine. (**a**) Schematic diagram. (**b**) Actual setup.

**Figure 4:**
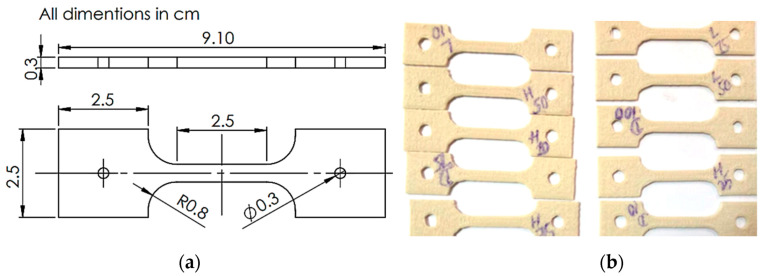
(**a**) Creep test specimen as per ASTM D2990 standards. (**b**) Printed creep specimens.

Figure 2 shows a schematic of the infill patterns considered in this study. A total of 39 experiments with coded parameters were conducted, based on Central Composite Design (CCD). These are presented in Table 3. This coding was adopted to simplify the calculation for regression analysis. Each specimen with a selected combination of process parameters is fabricated and tested. A creep testing machine is used to measure the creep rate of the FDM-manufactured parts under the constant stress of 15 MPa. Figure 3a,b shows the arrangements of the equipment and specimen clamping at the two points. The effect of temperature is not considered as it is well obvious from the previous studies. Therefore, the experiments are performed under constant room temperature, i.e., 25 °C. Table 3 shows the results of the creep rate and rupture time recorded for each test specimen. Figure 4a explains the geometry of the creep test specimen as per ASTM D2990 standards, and Figure 4b shows the actual printed test specimens developed for creep testing.

## 3. Results and Discussion

### 3.1. Model Fitting and Analysis of Variance

Analysis of variance (ANOVA) is performed to analyze the effect of input process parameters, i.e., layer height, infill percentage, and infill pattern on the responses such as creep rate (1/s) and rupture time (h) of PLA. To ensure the adequate prediction of responses compared to the experiments, the evaluation of a fitted model is essential. The prediction models of creep rate and rupture time based on CCD design for each infill pattern are expressed in Equations (1)–(6). Equations (1)–(3) represent the creep rate in terms of coded units, and Equations (4)–(6) represent the rupture time in terms of coded factors.

Diamond pattern = 0.109 − 0.001 L − 0.004 I − 0.004 L^2^ − 0.001 I^2^ − 0.001 L × I, (1)

Hexagonal pattern = 0.093 + 0.014 L − 0.004 I − 0.004 L^2^ − 0.001 I^2^ − 0.001 L × I, (2)

Linear pattern = 0.1094 + 0.001 L − 0.003 I − 0.004 L^2^ − 0.001 I^2^ − 0.001 L × I, (3)

Diamond pattern = 1.300 − 0.002 L − 0.052 I − 0.007 L^2^ − 0.025 I^2^ − 0.054 L × I, (4)

Hexagonal pattern = 2.14 − 0.025 L + 0.056 I − 0.008 L^2^ − 0.026 I^2^ − 0.054 L × I, (5)

Linear pattern = 1.305 + 0.005 L − 0.037 I − 0.008 L^2^ − 0.026 I^2^ − 0.054 L × I. (6)

ANOVA results are shown in Table 4. The second-order regression model for creep rate and rupture time is found at a confidence interval of 95%. Through ANOVA analysis, all the predicted coefficients are estimated with significant probability value, (*p*-value), i.e., *p* ≤ 0.05 and R^2^ (coefficient of determination) of 91% for creep rate, and 98% of rupture time, depicting higher validity to the predicted values for the two responses. The adjusted R^2^ coefficient value obtained for creep rate and rupture time is 87% and 97%, respectively. The coefficient values obtained confirm that the final prediction is in good agreement with the experimental results. Fisher’s statistical test (F-value) found for creep rate and rupture time were 22.48 and 206.1, which shows that the prediction is significantly useful. Likewise, *p*-values for lack of fit for creep rate and rupture time are higher than 0.05. From the observed statistical inferences, it was evaluated that the models accurately fit the experimental data. For the creep rate, the most significant terms are identified in which *p*-values are less than 0.05. These factors include the layer height, infill percentage, infill patterns, square of layer height and infill percentage, and interaction of layer height and infill pattern. For rupture time, the significant terms are the infill pattern type, the interaction of layer height and infill percentage, and infill pattern.

### 3.2. Regression Model Adequacy

**Figure 5:**
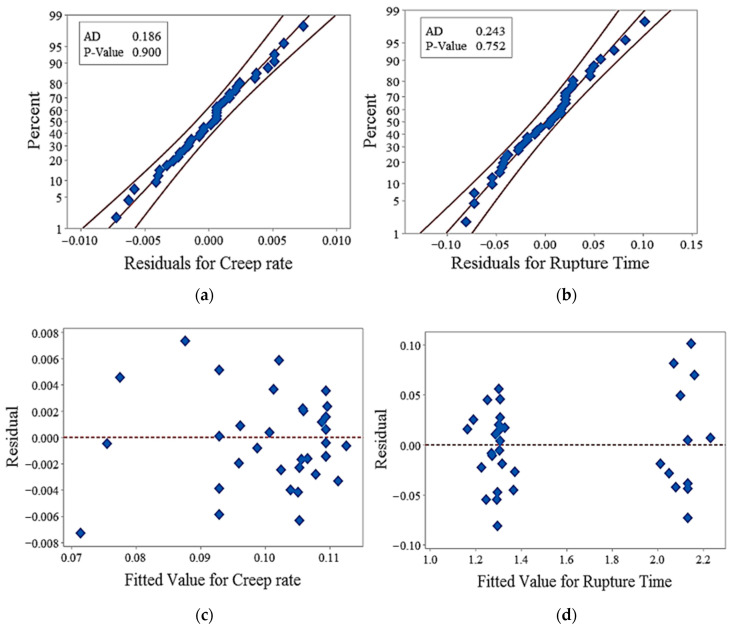
(**a**) Normality plot for creep rate. (**b**) Normality plot for rupture time. (**c**) Residual distribution for creep rate. (**d**) Residual distribution of rupture time.

The adequacy of the regression models is assessed through residuals normality and homoscedasticity. As shown in Figure 5a,b, the residuals for models falls near the continuous fitted line, so the normality of residuals for creep rate and rupture time is adequate. Anderson’s darling normality test further confirms that the residuals for creep rate and rupture time are normally distributed as the *p*-values are greater than 0.05, i.e., 0.900 and 0.752, respectively.

**Figure 6:**
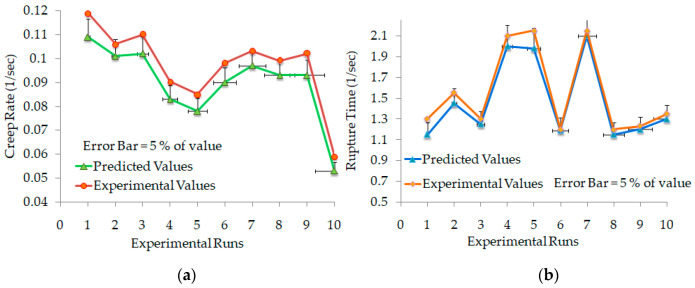
Experimental and predicted values. (**a**) Creep rate. (**b**) Rupture time.

In Figure 5c,d, the effect of residuals and fitted values for creep rate and rupture time are randomly distributed rather than forming the cone-shaped patterns in the distribution. This distribution shows that the residuals are homoscedastic and corroborate with the fitted value of creep rate and rupture time. To validate the prediction capability of the models, the predicted values of creep rate and rupture time for each type of infill patterns are compared with the experimental values, as shown in Figure 6a,b. The plots show that the predicted and experimental values are in good agreement with each other.

### 3.3. Effect of Process Parameters on Creep Rate and Rupture Time as 3D Surface Plots

**Figure 7:**
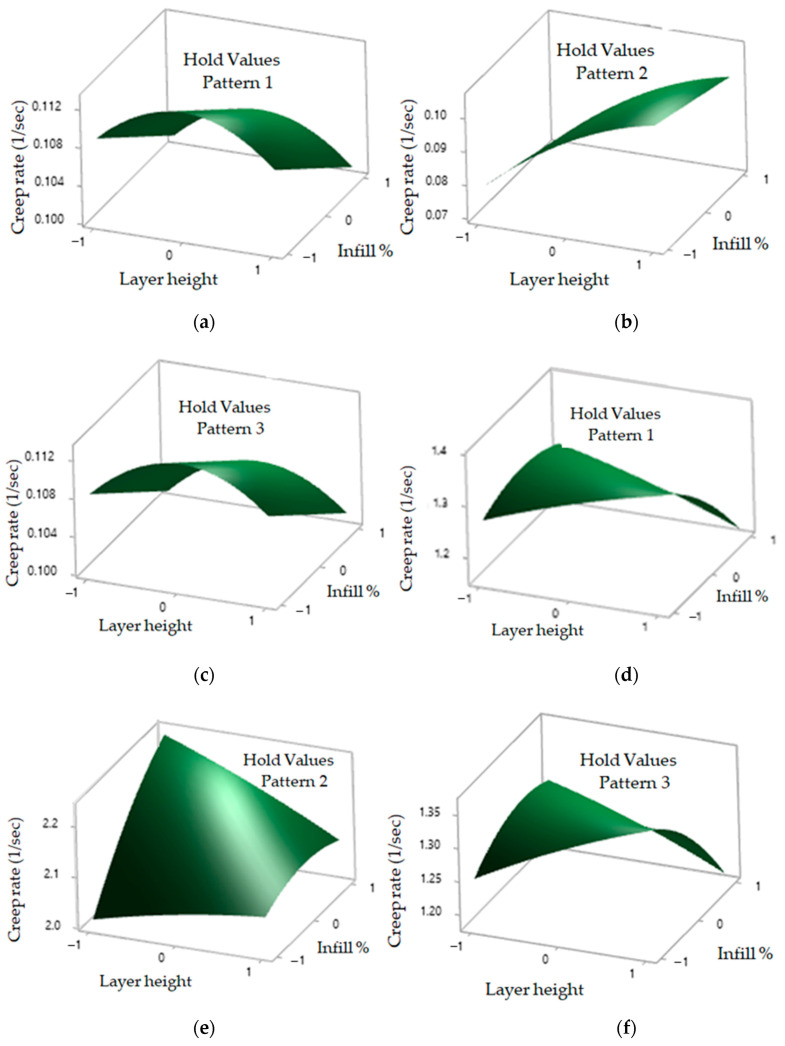
Interaction between layer height and infill %. (**a**) Creep rate of linear patterns. (**b**) Creep rate of hexagonal patterns. (**c**) Creep rate of diamond patterns. (**d**) Rupture time of linear patterns. (**e**) Rupture time of hexagonal patterns. (**f**) Rupture time of diamond patterns.

The effect of process parameters on creep rate and rupture time is observed in Figure 7 using 3D surface plots for linear, hexagonal, and diamond patterns. In Figure 7a, the creep rate for linear patterns increases as layer height increase from low level to the medium level, while it decreases as the layer height increase from a medium level to a high level.

The creep rate decreases with increasing the infill % from low level to high level. In Figure 7b, the creep rate for hexagonal patterns increases with an increase in layer height; however, for infill %, the creep rate decreases slightly from a low to a high level. In Figure 7c, similar results for the creep rate are observed in the diamond pattern. In Figure 7d, the rupture time for linear pattern increases slightly with an increase in layer height, while it decreases with an increase in the infill percentage from a low level to a high level. In Figure 7e, the rupture time for hexagonal pattern increases both in layer height and infill percentage. In Figure 7f, the rupture time for the diamond pattern shows similar plots for the linear pattern.

These results are in line with the published literature. Lower layer height provides good mechanical strength. As the layer height decreases, the bonding between the layers is strengthened and results in an improved mechanical performance [36,44]. A similar effect is observed for the infill percentage (the quantity of build material), where the mechanical strength improves with an increase in material density [45,46]. According to Dave et al. [35], the infill percentage of 100% results in strong bond formation between each layer, thus making the structure dense and stronger. However, with a decrease in infill %, the gap between printed layers becomes broader and reduces bonding strength. Strong bonding on six sides of the hexagonal pattern provides a strong contact with the neighbor molecules and, therefore, improves its tensile strength [47].

### 3.4. Multi-Response Optimization

**Figure 8:**
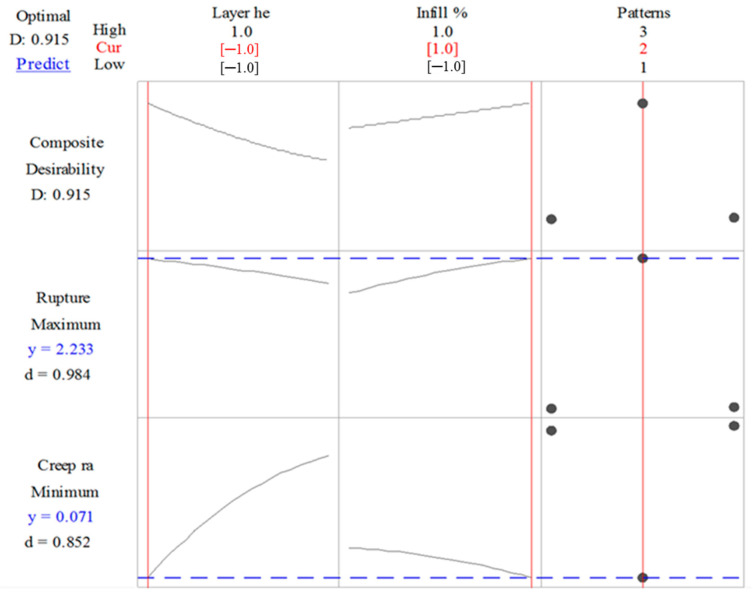
Multi-response optimization plot obtained for creep phenomenon using Minitab software.

The process parameters are optimized for rupture time and creep rate using composite desirability function. For each response, the criteria for the optimization set are different. For rupture time, the objective is to maximize the rupture time, while, for creep rate, the objective is to minimize the creep rate of PLA-printed samples. The multi-response optimization plot obtained using Minitab software is shown in Figure 8. The overall composite desirability function for creep behavior is 0.915, while the individual desirability values of rupture time and creep rate are 0.984 and 0.852, respectively.

The optimal combination of process parameters to increase the rupture time and reduce the creep rate of PLA printed parts are layer height at a low level (0.1 mm) and infill % at a high level (100%), while the desirable infill pattern is hexagonal. The predicted rupture time and creep rates are 2.233 h and 0.0712/s, respectively. Additionally, the plot shows that, by selecting the pattern type either 1 or 2, i.e., linear or diamond, the rupture time decreases, while the creep rate increases, which are both undesirable.

**Figure 9:**
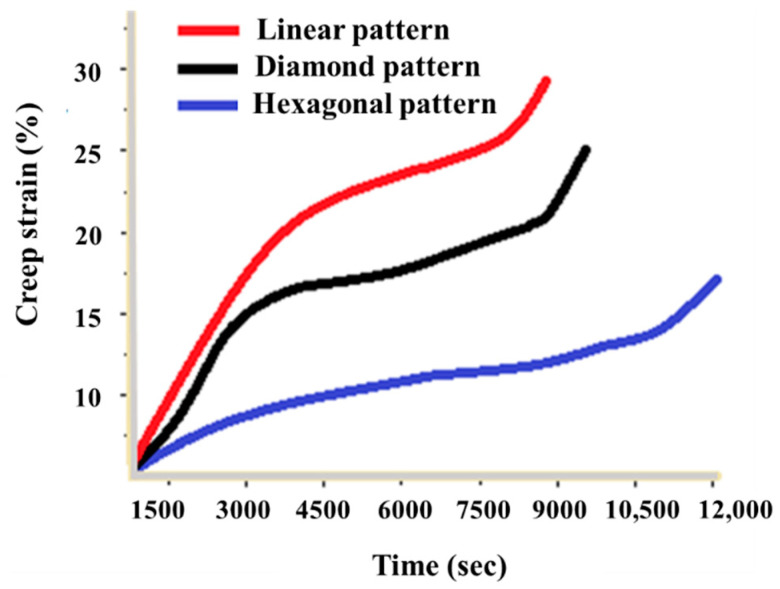
Time versus strain curve for creep testing specimens at different parametric setting.

To validate the performance of optimal parameters, confirmation tests were replicated five times. On average, the experimental values of rupture time and creep rate were 2.103 h and 0.0605 (1/s), respectively. The simulated values are close to the experimental results. Based on the experimental and statistical investigations, it can be concluded that the presented research provides a good guideline for the selection of categorical central composite design to analyze the creep behavior of PLA printed samples. Finally, the creep strain versus time is analyzed as shown in Figure 9 for all three types of patterns at 0.1 mm height and 100% infill percentage. The comparison shows that the creep strain in a hexagonal pattern is relatively lower, followed by diamond and linear pattern. This could be due to strong bonding on six sides of the hexagonal pattern providing a strong contact with the neighbor molecules. Farbman et al. [24] concluded that hexagonal infill pattern is stiffer and stronger than linear infill pattern. Lower layer height provides good mechanical strength. As the layer height decreases, the bond between the layers got strengthened, and, as a result, better mechanical strength is achieved [44]. Similarly, the infill percentage represents the quantity of build material as the density increases, and the mechanical strength also increases [44]. According to Dave et al. [35], the infill percentage of 100% results in strong bond formation between each layer, therefore making the structure dense and stronger. However, with a decrease in infill %, the gap between printed layers becomes broader and reduces bonding strength.

## 4. Conclusions

First, the present study optimizes the multi-response creep behavior of Poly Lactic Acid 3D Printed Parts using Response Surface Methodology. The ANOVA results show that all the predicted coefficients are estimated with significant *p*-value, i.e., *p* ≤ 0.05 and R^2^ of 91% for creep rate and 98% for rupture time. The adjusted R^2^ coefficient value obtained for creep rate and rupture time is 87% and 97%, respectively. The obtained results reveal that the final prediction is in good agreement with the experimental results. Likewise, F-values observed for creep rate and rupture time were 22.48 and 206.1, respectively, which shows that the prediction is significantly useful.

Second, the relationship between process parameters and responses were developed through successful prediction models. For linear and diamond patterns, the creep rate increases as the layer height increases from low level to the medium level, while it decreases as the layer height increases from medium level to a high level. By increasing the infill % from a low level to a high-level, a decrease can be seen in the creep rate. For a hexagonal pattern, the creep rate increases with an increase in layer height; however, for infill %, the creep rate remains almost constant from low to high level. For linear and diamond patterns, rupture time increases slightly with an increase in the layer height, while it decreases with an increase in infill % from a low level to a high level. For a hexagonal pattern, the rupture time decreases with an increase in infill% and layer height from a low level to a high level.

Third, the optimal combination of process parameters to increase the rupture time and reduce the creep rate of PLA printed parts are layer height at a low level (0.1 mm), infill % at a high level (100%), while the desirable infill pattern is hexagonal.

Fourth, the predicted rupture time and creep rates are 2.233 h and 0.071/s, respectively. To validate the performance of optimal parameters, confirmation tests are replicated five times. On average, the experimental values of rupture time and creep rate were observed as 2.103 h and 0.060 (1/s), respectively. The obtained results are close to the predicted values through statistical means. The experimental and statistical investigations provide good guidelines for the selection of categorical central composite design to analyze the creep behavior of PLA-printed parts (Figure 9).

Finally, the presented study also provides a comprehensive insight into the influence of FDM process parameters on creep rate and rupture time for the PLA 3D-printed products. This work is particularly interesting for small and medium enterprises in developing countries that are underpinned to adopt additive manufacturing by means of small-scale desktop 3D printers.

## Figures and Tables

**Table 1:** Polylactic acid (PLA) ((C_3_H_4_O_2_)_n_) properties.

**Mechanical Properties**	
Elastic (Young’s, Tensile) Modulus, GPa	3.5
Elongation at Break, %	6
Flexural Modulus, GPa	4
Flexural Strength, MPa	80
Tensile Strength: Ultimate (UTS), MPa	50
Molecular weight, M_n_ (g/mol) (10^3^)	4.7–16.8
**Thermal Properties**	
Heat Deflection Temperature At 455 kPa (66 psi), °C	65
Density, g/cm^3^	1.3
**Common Calculations**	
Stiffness to Weight: Axial, points	1.6
Stiffness to Weight: Bending, points	40
Strength to Weight: Axial, points	11
Strength to Weight: Bending, points	24

Note: PLA formula: n shows the repeating pattern of polymer.

**Table 2:** Factors with their levels.

Factors		Levels		
	Symbols	−1	0	1
Continuous				
Layer height (mm)	L	0.1	0.2	0.3
Infill percentage	I	10	55	100
Categorical		1	2	3
Infill Pattern	Pt	Linear	Hexagonal	Diamond

**Table 3:** Categorical Central Composite Design (CCD) matrix and measured responses.

Coded Variable					Responses	
Std Order	Run Order	Layerheight	Infill %	Patterns	Creep rate (1/s)	Rupture time (h)
27	1	−1	−1	3	0.108	1.296
16	2	−1	1	1	0.105	1.260
39	3	0	0	3	0.109	1.308
7	4	0	−1	2	0.094	2.021
10	5	0	0	2	0.087	2.088
30	6	1	1	3	0.101	1.212
18	7	−1	0	1	0.104	1.248
19	8	1	0	1	0.103	1.236
31	9	−1	0	3	0.101	1.212
25	10	0	0	1	0.110	1.320
11	11	0	0	2	0.089	2.136
1	12	−1	−1	2	0.082	1.994
34	13	0	1	3	0.099	1.188
12	14	0	0	2	0.098	2.058
22	15	0	0	1	0.110	1.320
13	16	0	0	2	0.093	2.093
21	17	0	1	1	0.100	1.200
14	18	−1	−1	1	0.105	1.260
3	19	−1	1	2	0.064	2.240
26	20	0	0	1	0.110	1.320
36	21	0	0	3	0.111	1.332
37	22	0	0	3	0.108	1.350
23	23	0	0	1	0.113	1.356
15	24	1	−1	1	0.112	1.344
8	25	0	1	2	0.095	2.233
2	26	1	−1	2	0.105	2.153
32	27	1	0	3	0.108	1.296
28	28	1	−1	3	0.110	1.320
20	29	0	−1	1	0.112	1.344
24	30	0	0	1	0.110	1.320
4	31	1	1	2	0.097	2.037
9	32	0	0	2	0.098	2.058
38	33	0	0	3	0.111	1.332
6	34	1	0	2	0.100	2.150
35	35	0	0	3	0.110	1.320
33	36	0	−1	3	0.108	1.296
17	37	1	1	1	0.098	1.176
29	38	−1	1	3	0.108	1.296
5	39	−1	0	2	0.075	2.250

**Table 4:** ANOVA of central composite design for creep rate (1/s) and rupture time (h).

Source	^†^ DF	Sum of Square	Mean Square	F-Value	^ǂ^*p*-Value
**Creep rate (1/s)**					
Model	11	0.003943	0.000358	22.48	<0.0001 *
L	1	0.000374	0.000374	23.43	<0.0001 *
I	1	0.000264	0.000264	16.59	<0.0001 *
Pt	2	0.002371	0.001185	74.34	<0.0001 *
L^2^	1	0.000128	0.000128	8.03	0.009 *
I^2^	1	0.00001	0.00001	0.63	0.436
L × I	1	0.000014	0.000014	0.88	0.356
L × Pt	2	0.000721	0.00036	22.6	<0.0001 *
I × Pt	2	0.000006	0.000003	0.2	0.821
Error	27	0.000431	0.000016		
Lack-of-Fit	15	0.000315	0.000021	2.17	0.091
Pure Error	12	0.000116	0.00001		
Total	38	0.004374			
**Rupture time (h)**					
Model	11	6.04684	0.54971	206.1	<0.0001
L	1	0.00098	0.00098	0.37	0.55
I	1	0.00192	0.00192	0.72	0.403
Pt	2	5.95786	2.97893	1116.89	<0.0001 *
L^2^	1	0.0004	0.0004	0.15	0.701
I^2^	1	0.00534	0.00534	2	0.169
L × I	1	0.03387	0.03387	12.7	0.001 *
L × Pt	2	0.00262	0.00131	0.49	0.617
I × Pt	2	0.04157	0.02079	7.79	0.002 *
Error	27	0.07201	0.00267		
Lack-of-Fit	15	0.06588	0.00439	2.2	0.097
Pure Error	12	0.00613	0.00051		
Total	38	6.11885			

Note: ^†^ DF is Degree of freedom, * means significant, ^ǂ^ Probability value
